# Comparison of sagittal measurements of cervical spine in spondylosis patients between Magnetic Resonance Imaging and Radiograph

**DOI:** 10.12688/f1000research.159504.1

**Published:** 2025-01-08

**Authors:** Maria Nicolet, Priyanka -, Rajagopal Kadavigere, Shailesh S Nayak, Saikiran Pendem, Surbhi Gupta Aggarwal, Tancia Pires, Varsha R

**Affiliations:** 1 Department of Medical Imaging Technology, Manipal College of Health Professions, Manipal Academy of Higher Education, Manipal, Karnataka, 576104, India; 2 Department of Radio diagnosis and Imaging, Kasturba Medical College, Manipal Academy of Higher Education, Manipal, Karnataka, 576104, India

**Keywords:** Cervical spine, Cervical spondylosis, Sagittal parameters, Magnetic Resonance Imaging, Radiograph

## Abstract

**Background:**

Cervical spondylosis is the common degenerative disease of the vertebrae in adults which can lead to change in sagittal alignment of cervical spine. Radiograph and Magnetic resonance imaging (MRI) are widely used imaging modalities for measuring the sagittal parameters. However sagittal parameters measured using radiograph and MRI can be influence by patient positioning and imaging technique. The study aims to compare sagittal parameters measured using MRI (Magnetic Resonance Imaging) and radiograph in cervical spondylosis patients.

**Methods:**

The study was done retrospectively. 77 patients who underwent both MRI and radiograph were included in the study. The sagittal parameters such as Neck Tilt (NT), T1 slope (T1S), thoracic inlet angle (TIA), C2-C7 angle (C2-C7A) and C2-C7 sagittal vertical axis (C2-C7 SVA) were measured on sagittal MRI and lateral cervical spine radiograph. Paired t-test was used to compare cervical sagittal measurements between MRI and radiography.

**Results:**

The cervical sagittal parameters such as NT, T1S, TIA and C2-7 SVA showed significant difference between MRI and radiograph (p < 0.05). But C2-C7A did not show significant difference (p > 0.05)

**Conclusion:**

The study concludes that MRI cannot be used as an alternative to cervical spine radiograph in spondylosis patient for measuring the sagittal balance as there was significant difference between sagittal parameters except C2-C7 A.

## Introduction

The cervical spine has the greatest range of motion in comparison to the rest of the spine and supports the weight of the head. Because of the intricacy of the cervical spine, it is prone to disease, including deformity. ^1^ Cervical spondylosis is the common degenerative disease of the vertebrae in adults. It is predicted that more than 85% of adults over the age of 60 will exhibit some evidence of cervical spondylosis on imaging tests, however all will not develop symptoms. Common cause of cervical spondylosis is aging, as we age the vertebral disc gets dissecated which reduces the spaces between the vertebral disc. This can result in pain, difficulty in movement and nerve damage. These degenerative changes can lead to the modification of cervical spine particularly the sagittal alignment. ^2– 4^

Sagittal alignment refers to the shape of the spine which is the sum of the shapes in the spine, discs, and muscle. Accurate measurement of cervical sagittal parameters such as thoracic inlet angle (TIA), neck tilt (NT) and T1 slope (TS) and sagittal vertical axis (SVA) are crucial for understanding the management of cervical spondylosis, eventually leading to better clinical results and higher patient quality of life. ^5– 7^

Radiographs (X-rays) and magnetic resonance imaging (MRI) are the imaging modalities used for measuring the sagittal parameters of c-spine. Radiographs are a rapid and cost-effective way to visualize bony structures and alignment, but MRI gives comprehensive details of both bony and soft tissue components, including intervertebral discs and the spinal cord. Despite their widespread usage, there may be inconsistencies in the sagittal parameters evaluated by radiography and MRI due to variances in patient posture, imaging procedures, and intrinsic modality-specific properties. ^4, 8, 9^

Currently few studies focus on the use of radiography or MRI individually for measuring the cervical sagittal parameters. However, no studies have directly compared the sagittal parameters between these two imaging modalities in cervical spondylosis. Such comparisons are required to assess if measurements from radiographs, which are more easily available in many clinical situations, can consistently replace those acquired from MRI.

## Methods

### Study design

This is a retrospective study. Approvals for the study was obtained from Institutional Research Committee (IRC) and Institutional Ethical Committee (IEC2-159-2022) of Kasturba Medical College and Hospital, Manipal, India on 12th May 2022. We used the STROBE reporting guidelines for our study; a completed checklist is available under Reporting Guidelines. ^10^ As this is a retrospective study, the data was collected from the patients who had undergone lateral radiograph in standing and MRI of C-spine in supine position within a time of one week between January 2016 to September 2022.

### Eligibility criteria

A total of 77 cervical spondylosis patients who had undergone lateral radiograph in standing and MRI of C-spine in supine position within a time of one week between January 2016 to September 2022 were included in the study. All the patients had undergone radiograph using computed radiography system (CR X-ray Wipro GE ^®, TM^) and MRI using Philips Achieva ^®, TM^ (1.5 T MRI). The sagittal T2 weighted MRI and lateral C-spine radiographic images were retrospectively retrieved from PACS (picture-archiving and communication system). The exclusion criteria included the following: patients with cervical spine surgeries, cervical spine deformity caused by fractures, tumour, infection or congenital disorders, neuromuscular disorder, spondylolisthesis and ankylosing spondylitis.

### Measurements of cervical sagittal parameters in radiograph and MRI

The cervical parameters such as NT, T1S, TIA, C2-C7 angle and C2-C7 SVA were measured on cervical lateral radiograph and sagittal T2 weighted MRI using measuring tool in PACS. All the measurements were done independently by the two readers and the average value was taken as final measurement.

**Figure 1.**
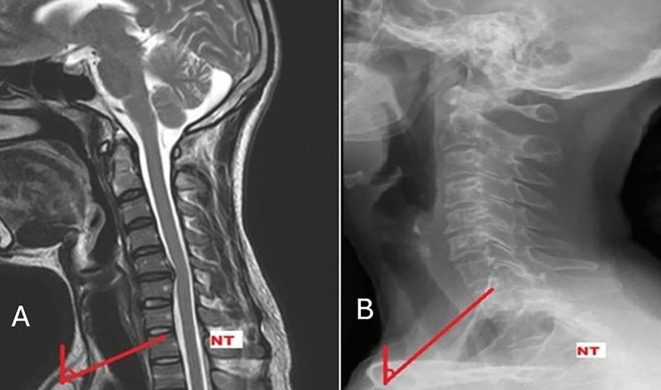
Measurement of neck tilt on MRI (A), and radiograph (B).

**Figure 2.**
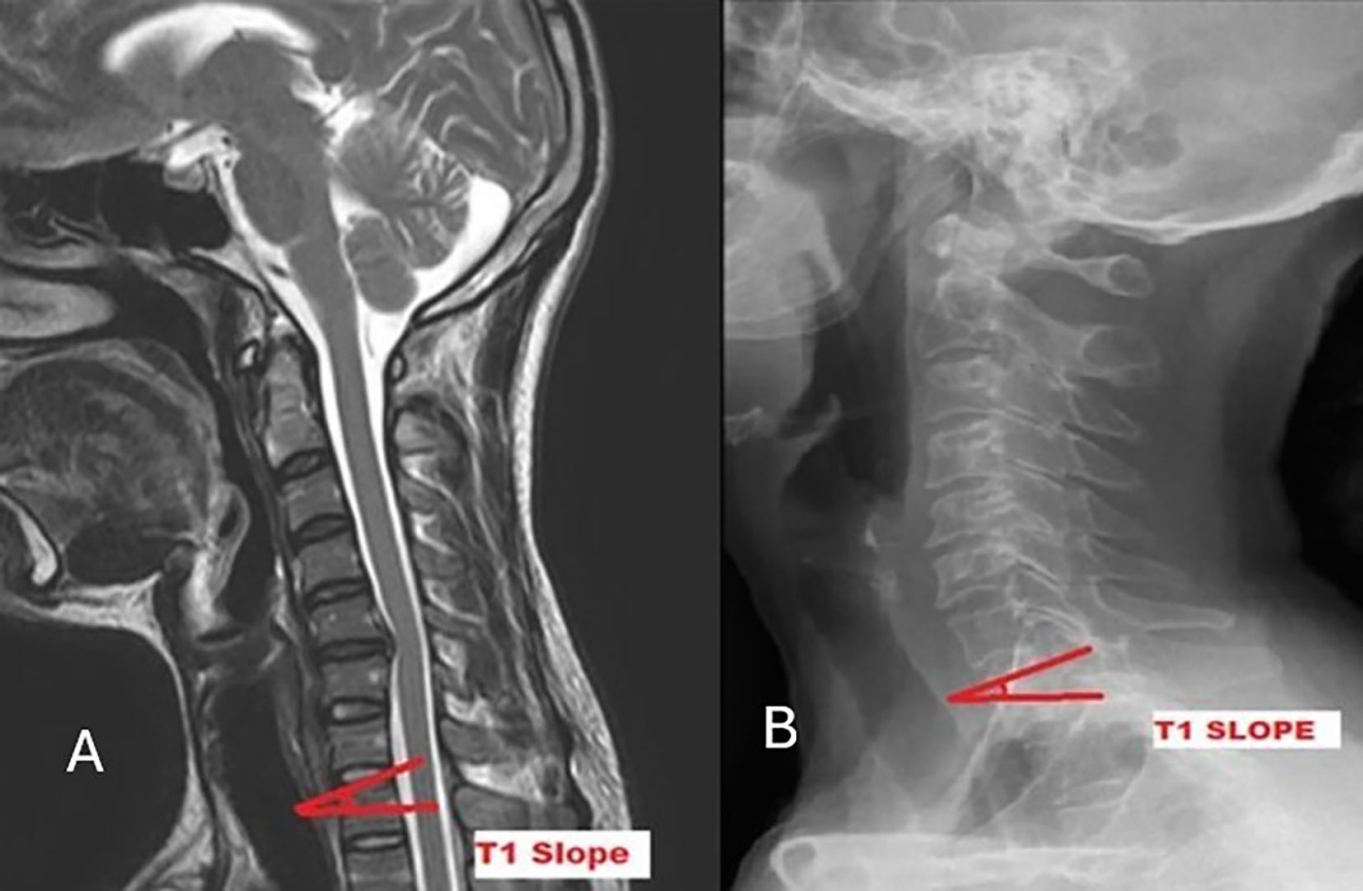
Measurement of T1 slope on MRI (A) and radiograph (B).

**Figure 3.**
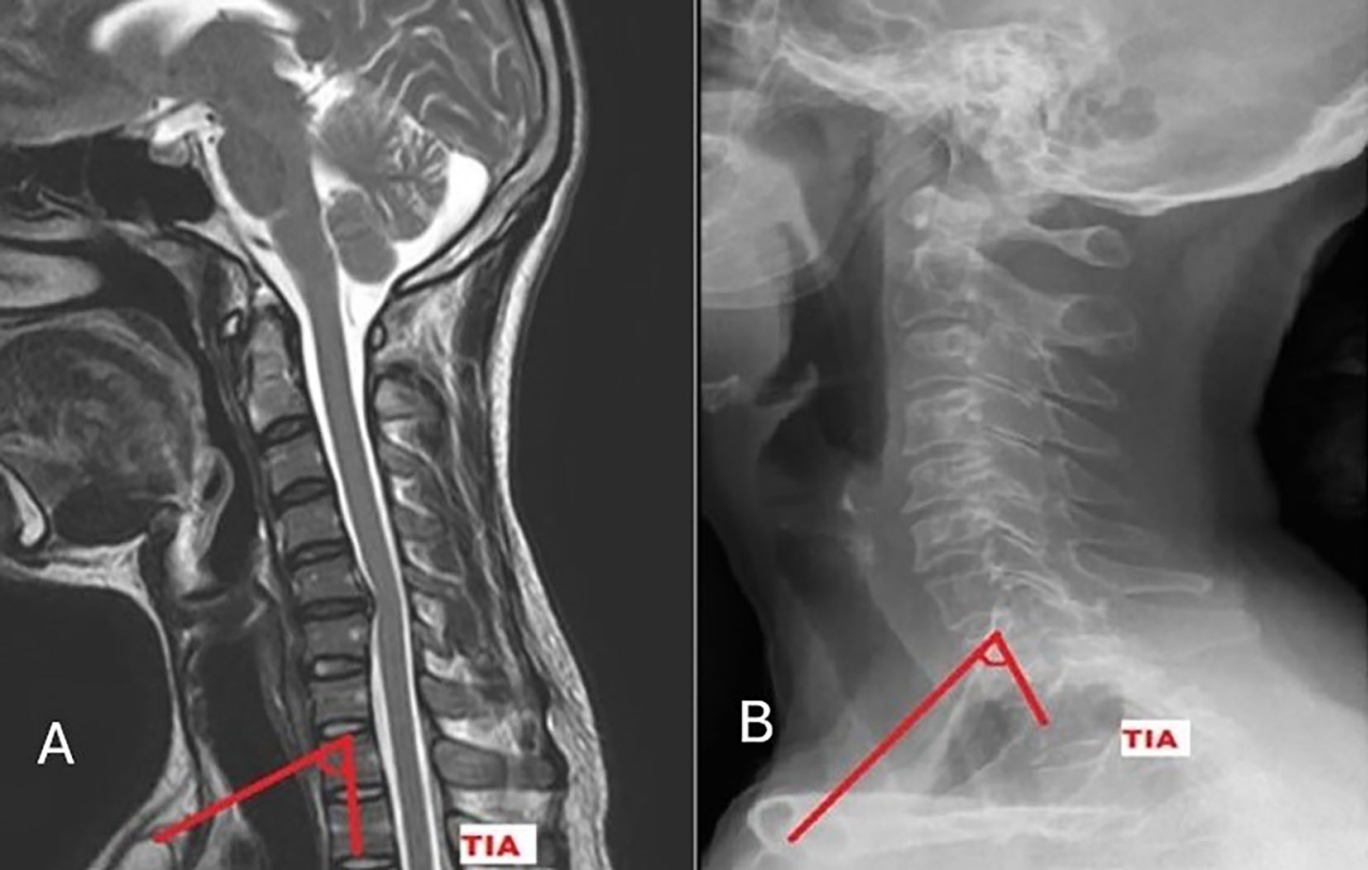
Measurement of Thoracic inlet angle on MRI (A) and radiograph (B).

**Figure 4.**
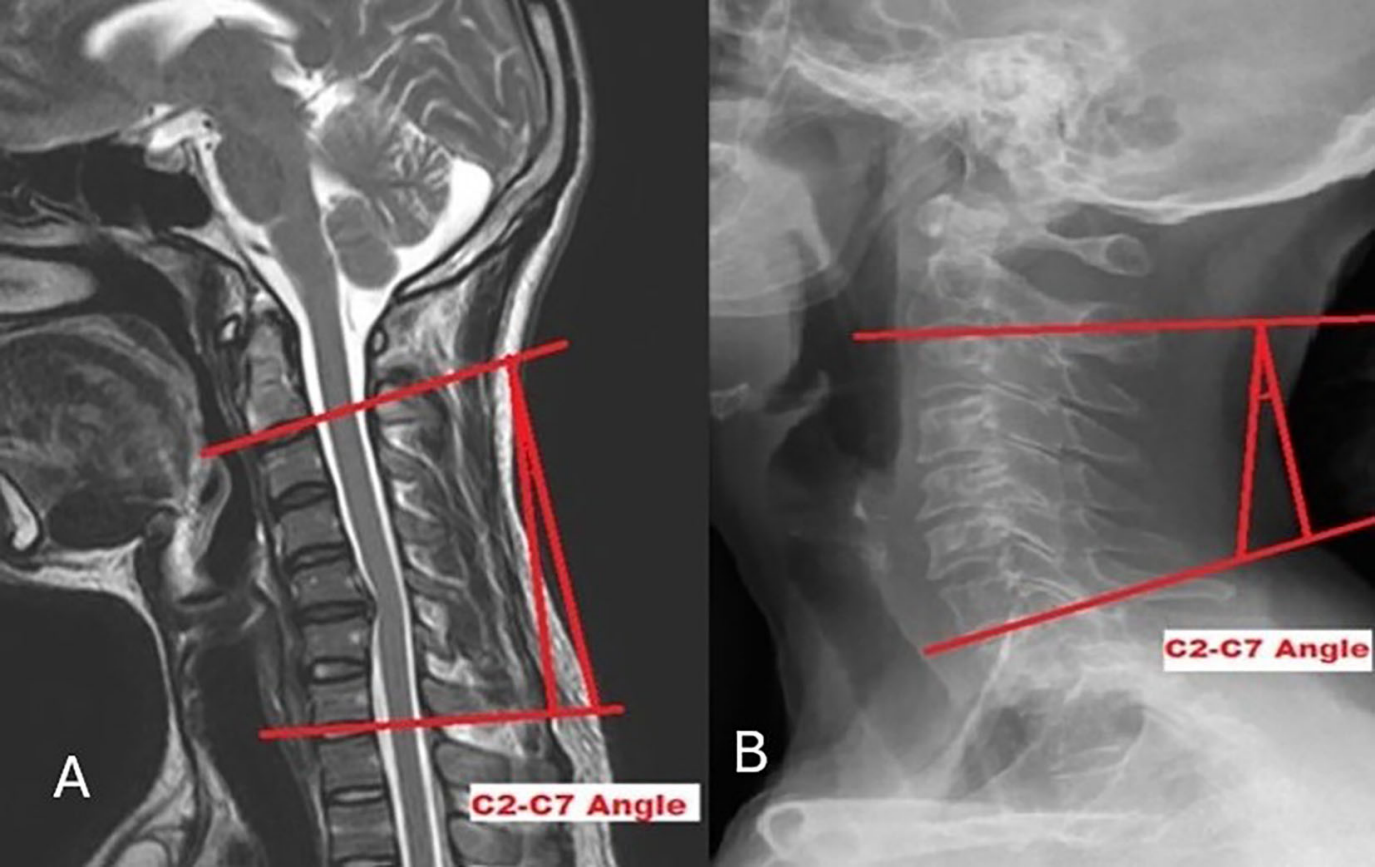
Measurement of C2-C7 angle on MRI (A) and radiograph (B).

**Figure 5.**
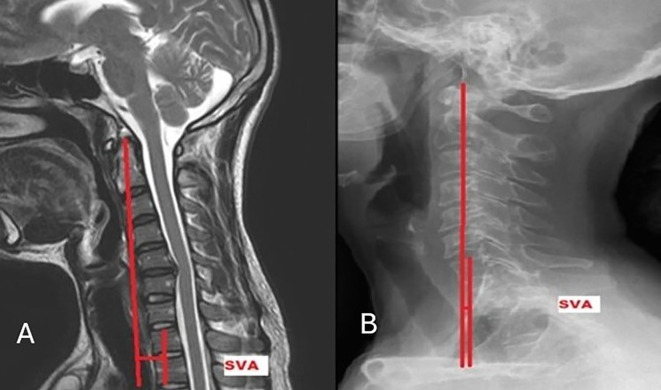
Measurement of C2-C7 sagittal vertical axis on MRI (A) and radiograph (B).

NT is the angle formed by “vertical line passing through the upper end of the sternum and a line connecting the centre of the first thoracic upper endplate (T1UEP) and the upper end of the sternum” ( Figure 1). T1S is the “angle formed between the horizontal plane and the T1UEP” ( Figure 2). TIA is the “angle formed by a line from the centre of the T1UEP vertical to the T1UEP and a line connecting the centre of the T1UEP and the upper end of the sternum” ( Figure 3). C2-C7A is the “angle between the horizontal line of the C2 lower endplate and the horizontal line of the C7 lower endplate” ( Figure 4). C2-C7 SVA is the “horizontal offset from the centre odontoid process (dens) to the centre of the vertebral body of seventh cervical spine (C7)” ( Figure 5). ^11^

### Statistical analysis

Statistical analysis of the data was performed using Jamovi-2.3.28.0 https://www.jamovi.org/. Paired t-test was used to compare cervical sagittal measurements between MRI and radiography. The ‘p’ value less than 0.05 was considered as significant (α = 0.05).

## Results

The study included a total of 77 patients out of which 39 of them were female and 38 were male who had undergone both C- spine MRI and lateral c-spine radiograph with the history of cervical spondylosis. The age was ranging from 25 to 71 years with mean age of the patient was 45 ± 11.22 years. The demographic details of the study participants are shown in Table 1.

**Table 1.** Demographic details of the study participants.

Demographic details (n = 77)	
Male (n)	38
Female (n)	39
Age in years (mean ± SD)	45 ± 11.22

SD standard deviation.

### Comparison of cervical sagittal parameters measured using MRI and radiograph

Cervical sagittal parameters such as NT, T1S, TIA, C2-C7 Angle and C2-C7 SVA was measured on MRI and X-ray image. Mean, standard deviation (SD) and difference in mean value of cervical parameters measured on MRI and radiograph was calculated and is shown in Table 2.

**Table 2.** Mean and standard deviation of cervical parameters measured on MRI and radiograph.

Parameters	MRI measurement (mean±SD)	Radiographic measurement (mean±SD)	Difference in measurements (MRI-radiograph) (mean±SD)	p- value
NT (degree)	53.91±11.61	49.31±8.67	4.59±9.6	<0.05
T1S (degree)	23.90±7.13	26.23±3.79	-2.32±7.75	<0.05
TIA (degree)	70.55±12.20	74.12±10.65	-3.57±12.09	<0.05
C2-C7A (degree)	15.37±5.86	15.87±7.52	-0.49±6.34	0.95
C2-C7 SVA (mm)	6.55±4.01	9.15±6.36	-2.59±6.59	<0.05

NT neck tilt, TIA thoracic inlet angle, T1S T1 slope, C2-C7 A C2 -C7 Angle, SVA Sagittal Vertical Axis, SD Standard Deviation.

Sagittal parameters such as NT (MRI: 53.91 ± 11.61; Radiograph: 49.31 ± 8.67), T1S (MRI; 23.90 ± 7.13; Radiograph: 26.23 ± 3.79), TIA (MRI: 70.55 ± 12.20; Radiograph: 74.12 ± 10.65) and C2-C7 SVA (MRI: 6.55 ± 4.01; Radiograph: 9.15 ± 6.36) showed statistically significant difference between MRI and radiograph (p < 0.05). However, C2-C7 A measurement did not show statistically significant difference between MRI (15.37 ± 5.86) and radiography (15.87 ± 7.52) ( Table 1).

## Discussion

The MRI and X-ray are the crucial imaging modalities used for the evaluation of degenerative changes in spine. In the present study the cervical sagittal measurements were compared between MRI and X-ray in cervical spondylosis patients. ^12, 13^ The following parameters such as NT, TIA, T1S, C2-7A and C2-7SVA were measured on both X-ray and MRI. The results of our study are compared with few other studies and the mean values are shown in the Table 3.

**Table 3.** Measurements of the cervical sagittal parameters of different studies.

Authors	Groups	NT (°)	T1S (°)	TIA (°)	C2-C7A (°)	C2-C7 SVA (mm)
Current study	MRI (Cervical spondylosis)	53.83 **±**11.4	23.80±6.9	70.45±12.4	15.36±6.3	6.42±3.9
	Radiograph (Cervical Spondylosis)	49.36±8.6	26.01±3.9	73.81±11	15.80±7.6	8.94±6.3
Xing et al., ^8^	MRI (Asymptomatic)	44.6±6.2	25.8±5.1	70.22±6.8	-	12.0±9.6
	MRI (Disc Degeneration)	48.6±6.8	22.9±7.0	71.5±8.0	-	16.7±11.9
Cheng et al., ^11^	MRI (Cervical spondylosis)	51.06±9.8	21.75±6.5	72.82±9.7	4.34±12.5	6.02±9.2
	Radiograph (Cervical spondylosis)	47.80±8.5	24.30±7	72.11±9.2	7.91±12	10.53±12.6
Lee et al., ^14^	Radiograph (Asymptomatic)	-	17.7±4.7	-	-	4.5±2.6
	Radiograph (Ankylosing spondylosis)	-	23.9±12.4	-	-	16.0±12.8
Xing et al., ^15^	Radiograph (Asymptomatic)	44.6±6.1	25.7±5.0	70.2±6.6	-	-
	MRI (Asymptomatic)	46.3±8.6	22.6±6.4	68.9±8.5	-	-
Zhang et al., ^17^	MRI (Cervical spondylotic myelopathy-Straightened group)	-	22.2±6.7	-	-	10.2±5.4
	MRI (Cervical spondylotic myelopathy-Lordosis group)	-	23.4±8.9	-	-	8.2±4.6
Park et al., ^16^	CT (Asymptomatic)	47.30±9.4	23.16±6.5	70.50±10.7	4.74±7.1	-
Yang et al., ^19^	MRI & radiograph (Cervical spondylosis)	-	-	76.07±9.5	-	21.34±11.4

TIA thoracic inlet angle, NT neck tilt,T1S T1 slope, SVA sagittal vertical axis.

Most of studies showed significant difference in sagittal parameters measured using MRI and radiograph which was similar to the findings of our study ( Table 3). Theses difference in measurement could be due to the position of the patient as MRI examination will be done with patients in supine position and radiograph will be done with patient in standing position.

In a study done by Lee et al., the mean value of T1S and C2-C7 SVA was higher compared to current study. However, in a study by Xing et al., the mean value of TIA, T1S and NT was lower compared to our study in MRI and radiograph. The difference between the mean values of the parameters in both the studies were since the values were measured on asymptomatic and our study had symptomatic patients. ^14, 15^ Bernstein et al., all segments of the MRI had better visibility between 70 and 100%, with the upper thoracic regions having the maximum visibility at 98 to 100%. Kyphosis angles could not be assessed in X-ray images because of the structures that obscured the higher thoracic structures, but no such issues existed in MRI. ^16^

Cheng et al., conducted a study in which NT, T1S, TIA, C2-C7A and C2-C7 SVA was measured both on MRI and radiograph. ^11^ Zhang et al., conducted a study, parameters such as T1S and C2-C7 SVA was measured in straightened group and lordosis group. ^17^ There is no disagreement on both the studies because the mean difference between their study and our study is remarkably similar. Park et al. investigated a study, the mean value of NT, T1S, TIA and C2-7A were measured. However, NT was higher in MRI in our study compared to their study. As MRI and CT are carried out in similar positions (supine), the mean of NT, T1S, TIA and C2-7A matched MRI but not radiograph. ^18^ Xing et al., the mean value of C2-C7 SVA was higher compared to our study and the NT, T1S and TIA value was similar to our study. ^15^ Yang et al. conducted a study on patients with neck pain, radiculopathy, and myelopathy. Measurements were done on MRI and radiograph. The mean of TIA and C2-C7 SVA were 76.07±9.54° and 21.34±11.42 mm respectively. ^19^ The mean value of TIA was almost similar compared to our study and the C2-C7 SVA was high compared to our study because in contrast to their work, the C2-C7 SVA that was assessed in our investigation was different.

The study has few limitations. The data was collected retrospectively, and therefore only limited patient’s clinical history was available. Secondly, the difference in measurement of sagittal parameters with age was not assessed. Future prospective studies can be performed with the age group classification.

## Conclusion

MRI cannot be used as alternative for C-spine radiograph for measuring the sagittal balance in cervical spondylosis patient as most of the sagittal parameters shows significant difference except for C2-C7 Angle **.** Therefore, standing C-spine lateral radiograph must be taken in patients with cervical spondylosis to measure the sagittal balance in addition to the MRI C-spine.

## Ethics and consent

This is a retrospective study. Ethical approval was obtained from the Institutional ethical committee (IEC2:159/2022) of Kasturba Medical College and Hospital, Manipal India on 12 ^th^ May 2022. This study aligns with the Declaration of Helsinki. The consent was wavied by ethical approval committee, as this is a retrospective study.

As this is a retrospective study, the data was collected from the patients who had undergone lateral radiograph in standing and MRI of C-spine in supine position within a time of one week between January 2016 to September 2022.

## Data Availability

Figshare: F1000 Data of Cervical Spine, https://doi.org/10.6084/m9.figshare.28080251.v1. ^20^ This project contains the following underlying data:
• X-ray images of C Spine • MRI images of C spine • X-Ray and MRI C spine sagittal measurements (Excel sheet) Data are available under the terms of the Creative Commons Attribution 4.0 International license (CC-BY 4.0). Figshare: We used STROBE checklist for “Comparison of sagittal measurements of cervical spine in spondylosis patients between Magnetic Resonance Imaging and Radiograph”. https://doi.org/10.6084/m9.figshare.28080371.v1. ^10^ Data are available under the terms of the Creative Commons Attribution 4.0 International license (CC-BY 4.0).
