# Supplementary material for: Comparison of sagittal measurements of cervical spine in spondylosis patients between Magnetic Resonance Imaging and Radiograph
Source: F1000Res. 2025 Jan 8;14:45. [Version 1] doi: 10.12688/f1000research.159504.1 (PMC11799758; doi:10.12688/f1000research.159504.1)
Supplement: Peer review history

# Reviewer report: Reviewer response for version 1

**DOI:** 10.5256/f1000research.175237.r358489

**Published:** 2025-02-05

**Authors:** Tarek Aly, Ahmed Aly

**Affiliations:** 1 Tanta University, Tanta, Gharbia Governorate, Egypt; 2 Internal Medicine, Tanta University Faculty of Medicine, Tanta, Gharbia Governorate, Egypt

Dear authors, thanks for giving me the opportunity to read your valuable manuscript. It is a very important subject which is still unclear in the literature. Some points in the manuscript needs to be clarified:

**Title**

 Radiography usually used for plain x-ray and CT. So, it is better to change to plain X-Ray.

**Results**

 Last line in results section: (Table 1) this table for demographics only!!>>>>>>> (Table2)

**Discussion**

 Line 6: similar to the findings of our study ( Table 3). Theses (These) difference …..

 You did not mention about the difference in measurements regarding the gender, and different age groups??

 Tables

 Table 1 did not add any information, so, it is better to be omitted.

Is the work clearly and accurately presented and does it cite the current literature?

Yes

If applicable, is the statistical analysis and its interpretation appropriate?

Yes

Are all the source data underlying the results available to ensure full reproducibility?

Yes

Is the study design appropriate and is the work technically sound?

Yes

Are the conclusions drawn adequately supported by the results?

Yes

Are sufficient details of methods and analysis provided to allow replication by others?

Yes

Reviewer Expertise:

spinal disorders

We confirm that we have read this submission and believe that we have an appropriate level of expertise to confirm that it is of an acceptable scientific standard.

# Reviewer report: Reviewer response for version 1

**DOI:** 10.5256/f1000research.175237.r358481

**Published:** 2025-01-23

**Authors:** Jaseemudheen MM

**Affiliations:** 1 Assistant Professor, Department of Medical Imaging Technology, K.S. Hegde Medical Academy, NITTE, Deralakatte, Mangalore, Karnataka, India. Pin: 575018, Karnataka, India

1. The full form of MRI is provided in the background (first part of the abstract). The repetition of MRI full form in brackets can be avoided in the same section. Mention the abbreviation alone.
2. The table number was provided wrongly (Last paragraph: Result section)
3. Check and correct the following sentence (Discussion): However, in a study by Xing et al., the mean value of TIA, T1S, and NT was lower compared to our study in MRI and radiograph. **The difference between the mean values of the parameters in both the studies were since the values were measured on asymptomatic and our study had symptomatic patients. ^14,15^**

Is the work clearly and accurately presented and does it cite the current literature?

Yes

If applicable, is the statistical analysis and its interpretation appropriate?

Yes

Are all the source data underlying the results available to ensure full reproducibility?

Yes

Is the study design appropriate and is the work technically sound?

Yes

Are the conclusions drawn adequately supported by the results?

Yes

Are sufficient details of methods and analysis provided to allow replication by others?

Yes

Reviewer Expertise:

Medical Imaging

I confirm that I have read this submission and believe that I have an appropriate level of expertise to confirm that it is of an acceptable scientific standard.
